# Safe and effective treatment of spontaneous neoplasms with interleukin 12 electro-chemo-gene therapy

**DOI:** 10.1111/jcmm.12382

**Published:** 2015-01-27

**Authors:** Jeffry Cutrera, Glenn King, Pamela Jones, Kristin Kicenuik, Elias Gumpel, Xueqing Xia, Shulin Li

**Affiliations:** a Department of Pediatrics, The University of Texas MD Anderson Cancer CenterHouston, TX, USA; b Department of Radiation Oncology, Gulf Coast Veterinary SpecialistsHouston, TX, USA

**Keywords:** electroporation, interleukin 12, chemotherapy, cancer, personalized therapy, immunotherapy

## Abstract

Electroporation improves the anti-tumour efficacy of chemotherapeutic and gene therapies. Combining electroporation-mediated chemotherapeutics with interleukin 12 (IL-12) plasmid DNA produces a strong yet safe anti-tumour effect for treating primary and refractory tumours. A previously published report demonstrated the efficacy of a single cycle of IL-12 plasmid DNA and bleomycin in canines, and, similarly, this study further demonstrates the safety and efficacy of repeated cycles of chemotherapy plus IL-12 gene therapy for long-term management of aggressive tumours. Thirteen canine patients were enrolled in this study and received multiple cycles of electro-chemo-gene therapy (ECGT) with IL-12 pDNA and either bleomycin or gemcitabine. ECGT treatments are very effective for inducing tumour regression *via* an antitumour immune response in all tested histotypes except for sarcomas, and these treatments can quickly eradicate or debulk large squamous cell carcinomas. The versatility of ECGT allows for response-based modifications which can overcome treatment resistance for affecting refractory lesions. Importantly, not a single severe adverse event was noted even in animals receiving the highest doses of chemotherapeutics and IL12 pDNA over multiple treatment cycles. This report highlights the safety, efficacy and versatility of this treatment strategy. The data reveal the importance of inducing a strong anti-tumour response for successfully affecting not only the treated tumours, but also non-treated metastatic tumours. ECGT with IL12 pDNA plus chemotherapy is an effective strategy for treating multiple types of spontaneous cancers including large, refractory and multiple tumour burdens.

## Introduction

Combining the cytotoxicity of chemotherapy with the anti-tumour immune responses of immunomodulatory therapy inhibits tumour growth in multiple types of cancer and even large, refractory tumours 1–3. Unfortunately, systemic administration of chemotherapeutics requires high doses with potentially dangerous side effects 4,5. Likewise, systemic delivery of immunomodulatory agents can create dangerous, robust immune responses while rarely resulting in a therapeutic effect 6,7. The potential therapeutic benefit for combining these treatments are drawing attention, but co-delivery methods are lacking. Thankfully, electroporation (EP) has emerged as a possible method for safely and effectively combining these treatments.

Electroporation is a physical method for transfecting agents, such as chemotherapeutics and plasmid DNA (pDNA), into cells. In EP, brief electric pulses induce transient pores in the cell membrane and transport the agents into the cytosol 8. EP does not cause any severe adverse events, and several veterinary clinical trials first showed the safety and efficacy of electrochemotherapy (ECT), chemotherapy delivered *via* EP 9–13. Indeed, the European Union has approved the use of EP with cisplatin and bleomycin for the treatment of certain cancer histotypes 8,14. Although these ECT treatments are successful, several shortcomings remain and alternative uses of EP may further improve the efficacy of EP-mediated treatments.

As a result of the high toxicity of delivering the recombinant cytokine, gene therapy with interleukin 12 (IL12) pDNA is another treatment strategy that can benefit from EP-mediated delivery to accessible tumour nodules (termed electrogenetherapy, EGT). Several Phase I trials using this exact treatment in melanoma and other histotypes have proven the safety, and Phase II studies are currently in progress to validate the efficacy. Additionally, the anti-tumour immune response induced by intratumoural treatment with IL12 pDNA can inhibit non-treated metastatic tumours 15, a shortcoming of ECT 8. Although ECT and EGT each are viable options for treating accessible tumours, combining these treatments into electro-chemo-gene therapy (ECGT) may be able to extend the efficacy of EP-mediated treatments while maintaining the safety seen with ECT and EGT 16.

A previous report presented the case results of five canine subjects that received only one intratumoural ECGT treatment cycle with IL-12 pDNA plus bleomycin 1,3. In that trial, the IL12 pDNA plus bleomycin ECGT treatments were able to completely eradicate squamous cell carcinoma (SCC) and acanthomatous ameloblastoma (AA) and resulted in at least a partial response (PR) in other histotypes. However, even in those with a complete response, the tumours can recur either from residual tumour cells in the same location or from micrometastasis which easily remain undiagnosed and untreated. Likewise, when tumours recur following treatment with any agent, treatment resistance can render ineffective the previously successful treatments. Therefore, it is important to determine whether repeated cycles of ECGT and/or alternating the chemotherapeutic agent can overcome this resistance while maintaining safety and tolerability.

To this end, this study was designed to allow for repeated treatment cycles, additional tumour histotypes and another chemotherapeutic agent (gemcitabine). The results from these studies will demonstrate the versatility, safety and efficacy of EP-mediated intratumoural IL12 pDNA treatments with and without chemotherapeutics.

## Materials and methods

### Patient selection

Over the course of 3 years, a total of 13 evaluable canines with naturally occurring neoplasms were enrolled in this study. The subjects' details are listed in Table1. The eligibility criteria included normal renal, hepatic and cardiac function along with diagnosed neoplasms accessible for direct injection and application of electric pulses. The subjects were staged with appropriate blood cell counts, serum profile, urinalysis, ECG and thoracic radiographs with CT scans as necessary to determine tumour invasion.

**Table 1:** Details of the enrolled subjects

Treatment	Response	Number of clinical responses per histotype				
		Responsive histotypes	SCC	AA	PC	Sarc
IL12 pDNA + Gem	PD	7 (32%)	7 (39%)	0 (0%)	0 (0%)	2 (66%)
	SD	7 (32%)	5 (28%)	1 (100%)	1 (33%)	1 (33%)
	PR + CR	8 (36%)	6 (33%)	0 (0%)	2 (66%)	0 (0%)
IL12 pDNA + Blm	PD	2 (13%)	1 (10%)	1 (25%)	0 (0%)	2 (100%)
	SD	5 (31%)	2 (20%)	3 (75%)	0 (0%)	0 (0%)
	PR + CR	9 (56%)	7 (70%)	0 (0%)	2 (100%)	0 (0%)
ECGT	PD	9 (24%)	8 (29%)	1 (20%)	0 (0%)	4 (80%)
	SD	12 (32%)	7 (25%)	4 (80%)	1 (20%)	1 (20%)
	PR + CR	17 (44%)	13 (46%)	0 (0%)	4 (80%)	0 (0%)
IL12 pDNA only	PD	0 (0%)	0 (0%)	0 (0%)	0 (0%)	0 (0%)
	SD	4 (44%)	3 (60%)	1 (25%)	0 (0%)	1 (100%)
	PR	5 (56%)	2 (40%)	3 (75%)	0 (0%)	0 (0%)
All Treatments	PD	9 (19%)	8 (24%)	1 (11%)	0 (0%)	4 (67%)
	SD	16 (34%)	10 (31%)	5 (56%)	1 (20%)	2 (33%)
	PR + CR	22 (47%)	15 (45%)	3 (33%)	4 (80%)	0 (0%)

### Treatment

All animal handling and treatments followed National Institute of Health (NIH) guidelines and were approved by the Institutional Animal Care and Use Committee at the University of Texas MD Anderson Cancer Center. Because of the lack of a commercially available canine IL12 pDNA, all previous veterinary studies in canines with IL12 pDNA have used either human or feline IL-12 pDNA constructs 17. To produce the canine IL12 used in this study, canine IL12 subunits P35 and P40 DNA were synthesized based on Genebank sequences NM_001003293 and NM_001003292 (GenScript USA) and subcloned to control vector pVC1157 under CMV promoter 18,19. All DNA were prepared using Endofree Plasmid Mega or Giga kits (Qiagen Sciences, Inc, Germantown, MD, USA). Prior to treatment, the pDNA was diluted to 2 mg/ml half-strength saline. Bleomycin (APP Pharmaceuticals, LLC., Schaumburg, IL, USA) and gemcitabine (Sagent Pharmaceuticals, Schaumburg, IL, USA) were purchased from the University of Texas MD Anderson pharmacy. The chemotherapeutics were diluted to 10 mg/ml with sterile saline prior to injection.

Prior to each treatment, a physical exam of the subject and an interview with the subjects' owner were performed. Anaesthesia was induced with intravenous propofol and maintained on isoflurane in oxygen. The details of the anaesthesia and other general veterinary practices were performed as per standard operating procedures as previously described 1. Once the subject was fully anaesthetized, the tumour was measured *via* callipers or a small millimetre ruler, as necessary for tumours in the oral cavity of small breed canines. At this point, biopsies from the tumour area were collected (after measurement and prior to treatment). With the IL12 pDNA and the chemotherapeutic diluted as described, the pDNA with or without chemotherapeutic was injected directly into the tumour tissue. For bleomycin the dose was 100 μl (1 unit) per centimetre, and the gemcitabine doses were increased in a dose escalation manner from 0.5 to 10 mg/cm (data not shown). None of these doses induced an adverse event and the optimal effective dose was determined to be 2 mg/cm of diameter. If the largest tumour diameter was greater than 1 cm, the tumour received an injection of the pDNA and chemotherapeutic for each centimetre (*e.g*. if the largest diameter is between 1 and 2 cm, the patient received two injections). Within 5 min. of the injections, a 6-needle electrode connected to a BTX 830 pulse generator was placed around the injection site and two 20-msec., 350 V/cm electric pulses with a 100-msec. interval between pulses were delivered 1,20–22. If multiple injections were performed, the same pulses were applied to each injection site. Each treatment was repeated at least one more time, and these two (or more) treatments composed a single cycle of treatment. At least once 2–3 weeks following the final treatment of each cycle, the subject was re-examined and the tumour volume measured. Based on the tumour response and the decision of the subject's owner, further treatments were performed.

### Clinical response measurement

At each visit, the tumour volume of all accessible tumours was measured *via* millimetre ruler or callipers. Two tumour diameters, the longest diameter (*a*) and the diameter perpendicular to the longest diameter (*b*), were measured, and the tumour volume (V) was calculated with the following formula: V + (π/8)*(a*b^2^). To analyse tumour growth in individual treatment cycles, the tumour volumes were normalized by the volume at the beginning of that cycle, so all day 0 values are 100%. Four (4) responses were calculated: complete response (CR), complete disappearance of all measurable nodules for at least 21 days with histopathological confirmation; partial response (PR), greater than 20% reduction from initial tumour volume prior to treatment cycle; progressive disease (PD), greater than 20% increase from initial tumour volume prior to treatment cycle; and stable disease (SD), neither significant volume increase for PD nor reduction for PR. These data were analysed *via* multiple *T*-tests at each time-point. Because of missing time-points, two-way ANOVA was not possible. Survival data were obtained by data mining the electronic medical record database at GCVS. Only patients with complete medical records from the day of cancer diagnosis until death or censored were included. Patients that received surgical interventions on the primary tumour were excluded as to only analyse standard, non-surgical treatments. The difference between survival curves was analysed with the Mantel-Cox test. Statistical analyses were performed with GraphPad Prism version 6.03 for Windows (La Jolla, CA, USA.)

## Results

### Subject attributes

A total of 13 evaluable subjects were enrolled in this study, and the details for the subject population are listed in Table1. In these 13 subjects, two had AAs, four had sarcomas and seven had SCC with one of these subjects developing a subsequent plasmacytoma. In total, 19 individual tumour nodules were treated, and these nodules involved the head and neck (*n* + 9), limbs (*n* + 7), and nasal plenum (*n* + 3). Only one patient had a distant metastatic nodule (SCC/PC subject). Nine of the thirteen subjects received previous treatment with radiation (*n* + 4), surgery (*n* + 3), or both surgery and radiation (*n* + 2). For all subjects that received previous treatment, disease progression resumed for at least 3 weeks prior to initiating any treatments in this trial. In these 19 nodules, a total of 58 treatment cycles were performed. Because of subject non-compliance, only 53 clinical responses could be determined. Details for the clinical responses are displayed in Table2.

**Table 2:** All clinical responses achieved by each treatment in each histotype

Subject information			Tumour Information			
Age (years)	Gender	Breed	Histotype	Location	Previous treatment	Best response
12	Male	Tibetan Terrier	AA	Oral (Mandible)	Radiation (2×)	PR
10	Female	German Shepherd	AA	Oral (Mandible)	None	PR
10	Male	Black Labrador Retriever	SCC/PC	Cutaneous (Limb)/Oral (Soft Pallete)	Surgery/None	CR/PR
9	Male	Chihuahua	SCC	Oral (Mandible)	None	SD
8	Male	Mixed	SCC	Oral (Maxillary Buccal)	Surgery + Radiation	CR
9	Male	Jack Russel Terrier	SCC	Oral (Retromolar Trigone)	None	PR
10	Female	German Shepherd	SCC	Oral (Lingual)	Surgery	PR
12	Male	Black Labrador Retriever	SCC	Nasal Plenum	Radiation (2×)	SD
9	Male	Yellow Labrador Retriever	SCC	Nasal Plenum	Radiation	SD
9	Female	Rough Collie	Sarcoma	Subcutaneous (Frontal Orbital)	Surgery + Radiation	PD
10	Male	Mix	Sarcoma	Subcutaneous (Limb)	Surgery	SD
12	Male	Dachsund	Sarcoma	Oral (Mandible)	None	PD
8	Female	Doberman Pinscher	Sarcoma	Subcutaneous (Limb)	Radiation (2×)	SD

The low number of patients and the heterogeneity among spontaneous neoplasms obfuscates statistical analyses; however, comparing the tumour volume changes within each individual treatment cycle (21–28 days) as separate curves allows multiple different analyses.

### Gemcitabine and bleomycin are both effective chemotherapeutics for ECGT with Interleukin 12 pDNA

Bleomycin and cisplatin are two chemotherapeutics that benefit from EP 23,24. Gemcitabine is another agent that could potentially benefit from EP because of its intracellular cytotoxic effects and low membrane permeability. Furthermore, the antitumour activity of gemcitabine is increased when the intracellular concentration is increased 25. Likewise, a preliminary murine study revealed that indeed gemcitabine plus IL12 pDNA are more effective for eradicating transplanted SCC tumours compared to the chemotherapeutic plus control pDNA (Fig. S1).

**Fig 1:**
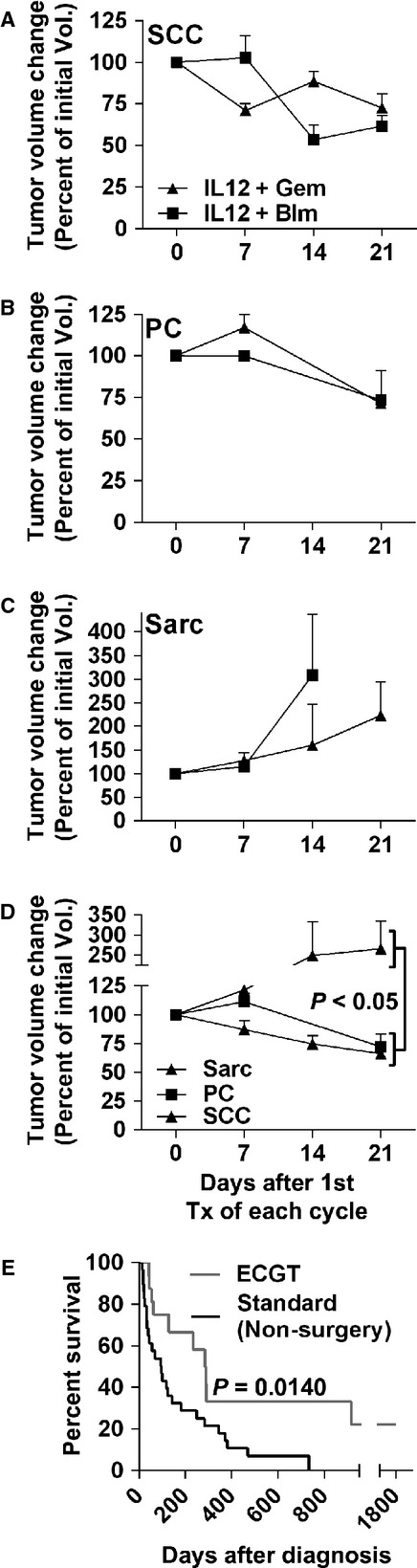
ECGT with IL12 pDNA is equally effective with gemcitabine or bleomycin. Analysing all treatment cycles with IL12 pDNA and either bleomycin (IL12 +  blm) or gemcitabine (IL12 +  gem) in squamous cell carcinoma (A, SCC, *n* + 9, 10), plasmacytoma (B, PC, *n* + 2, 3) and sarcoma (C, Sarc, *n* + 5, 4) reveals that ECGT is equally effective with blm or gem. (D) Comparison of all tumour histotypes shows that sarcomas do not respond to ECGT while PC and SCC are very responsive. (E) ECGT treatments extends survival compared to non-surgical standard treatments. Survival curve comparing ECGT (*n* + 16) and non-surgical standard treatments in subjects with complete records at the clinical trial host site. Error bars represent SEM.

The therapeutic benefits of ECGT with IL12 pDNA and bleomycin or gemcitabine were compared in multiple histotypes. Indeed, ECGT with bleomycin or gemcitabine are similarly effective in SCC and PC and equally ineffective for treating sarcomas. In oral and cutaneous SCC and PC, ECGT with IL12 pDNA plus either bleomycin or gemcitabine reduced tumour volume by 21 days after the first treatment of that cycle (Fig.1A and B). Notably in SCC, the IL12 pDNA plus bleomycin treatments have a more immediate effect (within 7 days) while the IL12 pDNA plus gemcitabine is slightly slower (within 14 days). In PC, only five total treatment cycles (two with gemcitabine and three with bleomycin) were performed, yet the curves are very similar and all result in an average of 27% reduction of tumour volume in 21 days (Fig.1B). Conversely, one histotype, sarcomas, did not respond to these treatments and both ECGT with bleomycin or gemcitabine failed to inhibit tumour growth (Fig.1C). Pooling the ECGT data and comparing the tumour growth curves across the histotypes revealed a significant difference between the 27% volume reduction in responding histotypes, SCC and PC, and the 165% volume increase in non-responding sarcomas (Fig.1D).

### IL12 boosts the antitumour immune response in normal, non-immune-privileged tissues

**Fig 2:**
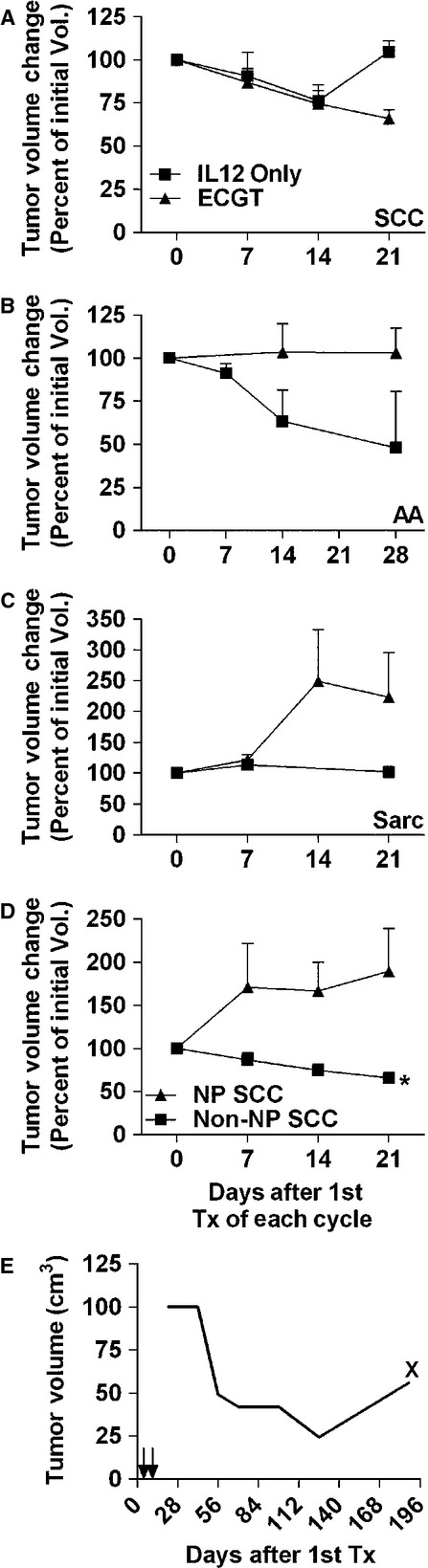
IL12 pDNA can confer an antitumour immune response. (A) In squamous cell carcinoma, ECGT with IL12 pDNA and a chemotherapeutic induces tumour regression equally compared to IL12 pDNA alone plus electroporation, but the tumour growth resumes by day 21 after treatment (*n* + 5 for IL12 pDNA alone and *n* + 20 for ECGT). (B) In acanthomatous ameloblastoma, IL12 pDNA alone plus electroporation appears more effective than ECGT with IL12 pDNA and a chemotherapeutic, although the difference is not significant (*n* + 4). (C) In Sarcomas, IL12 pDNA alone plus electroporation (*n* + 2) appears to stabilize the tumour volume while ECGT with IL12 pDNA and a chemotherapeutic (*n* + 9) cannot inhibit tumour growth; however, the low sample size of IL12 pDNA prohibits statistical analysis. (D) SCC tumours located anywhere except the nasal planum (non-NP SCC, *n* + 20) respond to ECGT with an average 30% tumour reduction in only 21 days, but nasal planum SCC tumours (NP SCC, *n* + 8) do not respond to ECGT. (E) A non-treated metastatic lymph node SCC tumour steadily regressed after two treatments in the initial dorsal carpus tumours on days 0 and 7. At 96 days after the treatments in the dorsal carpus tumours, progression of the metastatic nodule was noted. X denotes surgical removal of the metastatic tumour. Error bars represent SEM.

To ensure that the gene component of the ECGT treatment is conferring a benefit, some patients received EGT with only IL12 pDNA. In cutaneous and oral SCC, EGT and ECGT treatments equally regressed tumour volume by 25% through day 14; however, by day 21, tumour growth resumed in those receiving IL12 pDNA only while tumour volume continued to decrease in those patients receiving ECGT (Fig.2A). On the other hand, AA lesions seemed to respond better to the EGT treatments, although the small sample size and large variation did not confer significance (Fig.2B). Interestingly, sarcomas, in which ECGT failed to inhibit tumour growth, appeared to stabilize following IL12 only treatments, but again, low sample size prevented statistical analysis (Fig.2C). Alone, these results demonstrate that EP-mediated IL12 pDNA EGT treatments can inhibit tumour growth.

Three SCC lesions on the immune-privileged nasal planum received treatment with ECGT, but these treatments were incapable of inhibiting tumour growth. Compared to the 27% average regression from ECGT treatment in oral and cutaneous SCC, nasal planum SCC lesions did not respond to ECGT treatments and tumour volume increased almost 75% in 21 days (Fig.2D).

In one patient with SCC lesions, a metastatic SCC lesion was identified in the pre-scapular lymph node ipsilateral to the tumour-bearing forelimb 21 days after the first treatment cycle began. This tumour did not receive any intratumoural treatments, yet the volume of this lesion regressed steadily for nearly 120 days after the only treatment cycle performed in the forelimb SCC lesions (Fig.2E). On day 189, an increase in volume of the metastatic lesion was noted, and the owner elected for surgical removal of the affected lymph node. The recurring growth suggests that the antitumour immune memory was lost or no longer sufficient to inhibit growth of this metastatic lesion. Together, these data further provide evidence that IL12 in these treatments induces an antitumour response in normal tissues. The lack of response to ECGT in immune-privileged tissue (*i.e*. nasal planum) indicates that direct cytotoxicity by ECT is not sufficient to induce the robust anti-tumour response seen in ECGT.

### Treatment with EP-mediated IL12 alone or with chemotherapy is effective in large and small tumours

**Fig 3:**
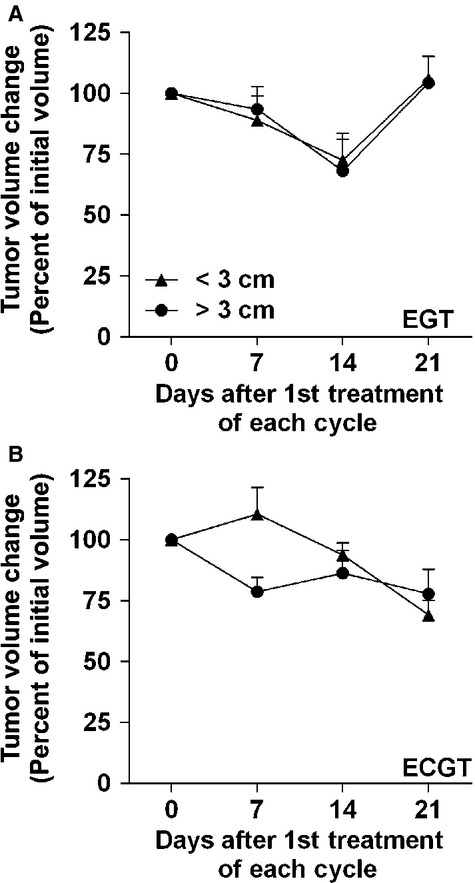
IL12 pDNA alone or with chemotherapy can equally affect large and small tumours. (A) EP-mediated EGT (IL12 pDNA alone) equally regresses small (<3 cm, *n* + 4) and large (>3 cm, *n* + 5) tumours for 14 days, but tumour progression returns by day 21. (B) ECGT (IL12 pDNA plus either bleomycin or gemcitabine) equally regresses small (<3 cm, *n* + 11) and large (>3 cm, *n* + 19) tumours. Error bars represent SEM.

The efficacy of most cancer treatments is inversely proportional to the size of the tumour, *i.e*. the smaller the tumour, the more effective the therapeutic. The efficacy of these treatments based on tumour size was determined by pooling all treatment cycles from responding histotypes and then separating into groups based on initial tumour diameters < or >3 cm. In IL12-only EP-mediated treatments, there are no differences in response compared to ECGT with tumour volumes being reduced by at least 27% on day 14 after both treatments; however, tumour growth returned in the IL12-only treatment group to the initial volume by day 21 (Fig.3A). Similarly, ECGT treatments regressed both small and large tumours more than 10% on day 14 and more than 20% on day 21 (Fig.3B). As also seen in Figure3, the IL12 only treatments can reduce tumour volume, but the addition of chemotherapy extends that antitumour effect.

### ECGT with IL12 and gemcitabine or bleomycin can eradicate or debulk large SCC tumours

**Fig 4:**
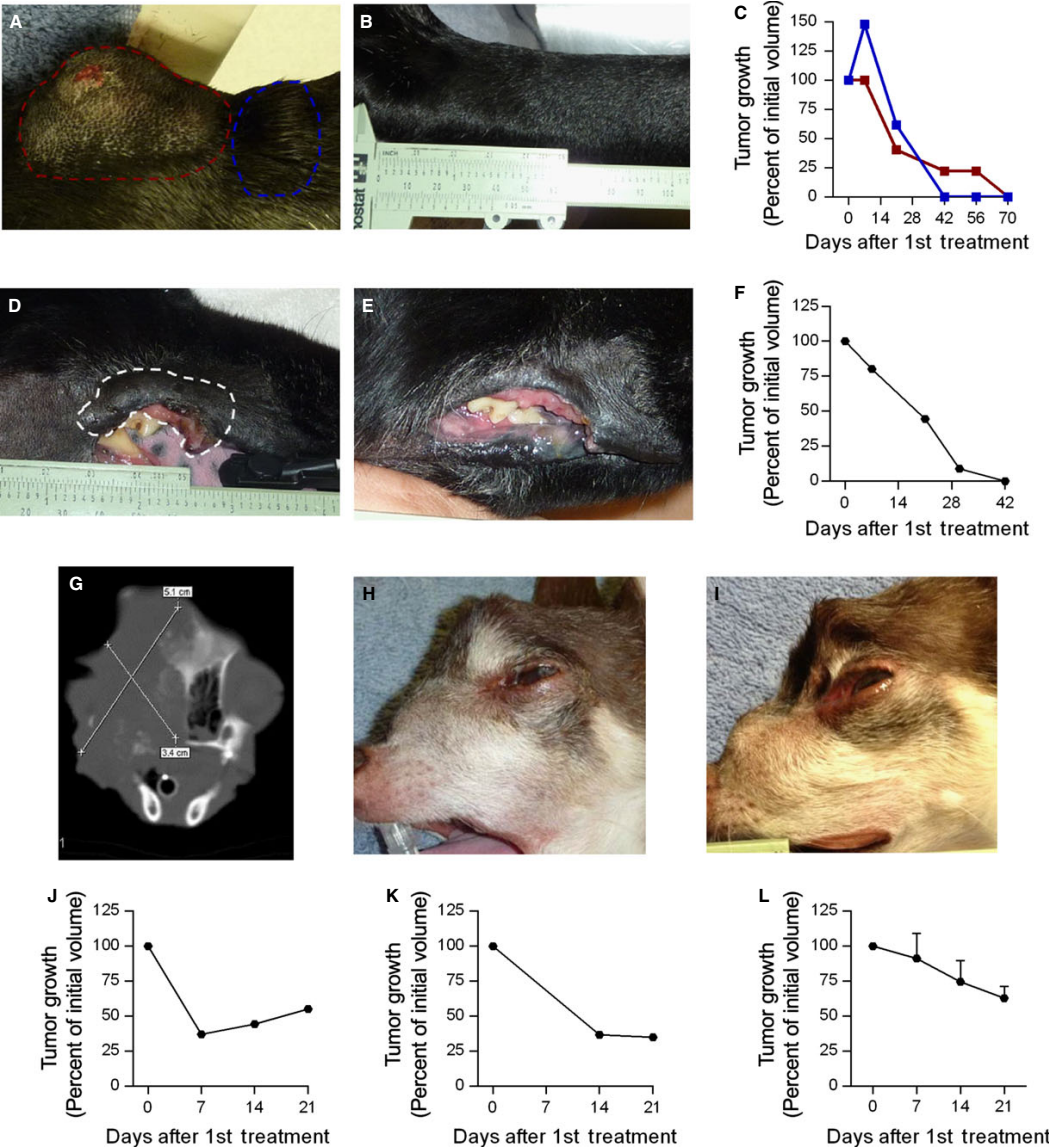
ECGT with IL12 pDNA and either bleomycin or gemcitabine can eradicate or debulk large squamous cell carcinoma tumours. (A) Squamous cell carcinoma tumours on Day 0 prior to ECGT with IL12 pDNA and bleomycin. The larger tumour is outlined in blue and the samller tumour is outlined in red. (B) Eradicated tumours on Day 70. (C) Tumour volume curves showing the eradication as per cent of tumour volume on Day 0. The coloured lines corresponds to the tumours outlined in A. (D) Maxillary Squamous cell carcinoma tumours on Day 0 prior to ECGT with IL12 pDNA and gemcitabine. (E) Healthy maxillary tissue with no evidence of residual SCC tumour cells as determined by histopathological analysis. (F) Tumour volume curve showing the eradication as per cent of tumour volume on Day 0. CT scan (G) and photograph (H) of an invasive squamous cell carcinoma tumour on Day 0 prior to ECGT with IL12 pDNA and bleomycin. (I) Photograph illustrating quick debulking of SCC on day 7 after only 1 ECGT treatment. (J) Tumour volume curve showing tumour reduction as per cent of tumour volume on Day 0. (K) Tumour volume curve showing tumour reduction as per cent of tumour volume on Day 0 of a subject with an SCC tumour in the retromolar trigone after only 1 ECGT treatment with IL12 pDNA and bleomycin. (L) Average tumour volume reduction in SCC tumours with ECGT with IL12 pDNA and either bleomycin or gemcitabine. Error bars represent SEM (*n* + 8).

Squamous cell carcinoma was the most prevalent histotype enrolled in this trial. In concert with our previously published study 3, large and small SCC tumours (longest tumour diameter >3 cm and <3 cm, respectively) can be eradicated with one treatment cycle of IL12 pDNA plus bleomycin ECGT (Fig.4A–C). Similarly, one cycle of two IL12 pDNA plus gemcitabine (2 mg dose) ECGT treatments eradicated in only 42 days a 3.2-cm maxillary buccal SCC tumour in a different patient (Fig.4D–F).

These remarkable complete responses are not always the case with SCC lesions; however, the power of the ECGT treatments repeatedly induces rapid tumour regression, although not always to a complete eradication. For instance, another patient presented with a large invasive maxillary SCC (Fig.4G and H) which distorted the facial features to the point that sight was lost in the left eye. Seven days after only one IL12 pDNA and gemcitabine ECGT treatment, this tumour was reduced by 63% (Fig.4I and J). As a result, the eye moved back into place and sight returned (Fig.4I). Similar regression was seen in another patient with an SCC lesion in the retromolar trigone after receiving IL12 pDNA plus bleomycin (Fig.4K). In all patients with either cutaneous or oral SCC (*n* + 8), the first treatment cycle of ECGT, with gemcitabine or bleomycin resulted in an average decrease to 63% of initial tumour volume after only 21 days (Fig.4L). So, ECGT can be used as a first-line treatment for cutaneous and oral SCC to reduce tumour volume in sensitive, vital areas or to rapidly debulk tumour volumes for increased efficacy of subsequent treatments, such as surgery or radiation.

## Discussion

The potential for inducing an antitumour immune response to treat cancers is highly published; however, the application of this technology has yet to be meaningfully transferred into the clinic. Throughout the relatively brief history of immune modulatory therapies, only minor successes in clinical cancer treatments have been achieved while several severe adverse events including deaths have occurred 7,26,27. Local administration of recombinant cytokines directly into the tumour has proven to be much safer than systemic delivery; however, delivering the gene encoding the cytokine has proven to be much more effective for maintaining safe and therapeutically relevant levels of the cytokine 28. A recent study demonstrated that only 0.1% of cells in the tumour environment need to express IL-12 to confer an anti-tumour immune response, so the gene only needs to be transfected into a small portion of the tumour cells 29. One method for obtaining these goals is EP, the application of specific electric pulses directly to the tumour after intralesional injection of the pDNA.

Electroporation is a versatile method which can be used on almost all cell types *in vitro* and tissues *in vivo* 30. Although the mechanism is not completely understood, EP occurs through a five-step process: charging, induction, expansion, stabilization and resealing 31. These five steps are initiated by the electric pulses, and several different sets of parameters can induce similar EP in the same type of tissue. For instance, several groups have successfully used very high-voltage (>1300 V/cm) parameters with many (6–8) short pulses (50–100 μsec.) for delivery of pDNA or chemotherapeutics 15,32,33. Likewise, the low voltage, long-duration parameters of two 20-msec. pulses with 350 V/cm used in this study have also been successfully employed for EP-mediated delivery of chemotherapeutics (ECT), pDNA (EGT) and the combination (ECGT) 1,16,21,22,30. Alternatively, a new branch of EP termed irreversible EP can induce direct cell death with much higher voltages, more pulses and other parameter modifications 34,35. EP is an adaptable method which will continue to be improved and utilized in the future to improve delivery and efficacy.

Interleukin-12 is a naturally occurring cytokine that continues to show potential success for treating cancer by inducing specific anti-tumour immune responses. Following the initial success of IL12 studies in preclinical models, Phase I human clinical trials appeared to confirm the safety of delivering IL12 recombinant protein (rIL12). Sadly, the same dose level of rIL12 that appeared safe in the Phase I trials caused severe side effects including death, and the therapeutic responses seen in murine models were not repeated in humans 26,36. These results, along with the short half-life of the IL-12 cytokine, preclude IL-12 treatments with recombinant proteins.

Like the recombinant protein studies, gene therapy with IL12 pDNA is consistently shown to effectively treat multiple tumour histotypes in rodent models and induce an anti-tumour immune response capable of affecting non-treated, metastatic nodules 27,36–39. Likewise, IL12 pDNA treatments in larger animals such as equine 38,40,41 and canine 6,11 models can induce tumour regression in target (*i.e*. treated) and non-target (*i.e*. non-treated) spontaneous melanoma nodules, as well as other tumour histotypes 6,17,42. In this study, intratumoural IL12 pDNA treatments induced tumour regression in large AA and SCC tumours (Fig.2A and B). Importantly, tumour growth did eventually resume in some patients, in concert with results seen in other studies 38, indicating that the anti-tumour immune response induced by IL12 is only transient. These results and many more warrant further study in humans, and, presently, there are numerous human clinical trials investigating the safety and efficacy of IL-12 gene therapies. These studies have shown that IL-12 can be safely administered as a gene, but the therapeutic successes in humans remain difficult to achieve 15. According to the FDA website at the time of publication, the Center for Biologics Evaluation and Research has not approved any human gene therapies in the United States 23 years after the first gene therapy clinical trial.

Since EP is not specific for only pDNA, a nearly innumerable amount of agents including chemotherapeutics can be delivered to tumours, as well as almost any other tissue 43. As seen in gene treatments, EP can significantly increase by 1000-fold the efficacy of several chemotherapeutics with poor cell permeability and internal targets, such as bleomycin and cisplatin 23,24. Indeed, cisplatin was first used with EP in veterinary patients over 10 years ago 44, yet very few veterinary clinics, only three in the United States, offer ECT as a treatment even though studies continue to show the benefits for companion animals 33,45.

With this system, almost any chemotherapeutic with poor membrane permeability that requires internalization could become more effective if delivered in conjunction with EP. One such agent is gemcitabine, a pyrimidine analogue which inhibits DNA synthesis but has difficulty entering cells to induce tumour cell death. Indeed, the efficacy of gemcitabine for treating pancreatic cancer can be improved with the co-administration of Nanoparticle albumin-bound (nab)-paclitaxel. The nab-paclitaxel created increased intracellular concentrations of gemcitabine *via* inhibition of cytadine deaminase, a gemcitabine metabolizing enzyme 25. So, the efficacy of gemcitabine should similarly increase with EP by increasing the internal concentration.

Recently, the European Union approved ECT, EP with single chemotherapeutic agents, based on the success of clinical trials. However, as was seen in murine models 16, the success of ECT for obtaining an objective response depends on the size of the largest tumour diameter, with larger tumours responding much less frequently than those with smaller tumour diameters 46. ECT has been effective for smaller primary tumours or as an adjuvant therapy, but resistance, size and metastasis, three major factors that contribute most to cancer deaths, continue to hinder the success of ECT.

Although both IL12 pDNA and chemotherapeutic treatments with EP have been shown to be effective, the synergistic power of combining these treatments may be able to overcome the above listed shortcomings of either treatment alone. In a murine study, intratumoural ECGT with IL12 pDNA and bleomycin was much more effective compared to either treatment alone for inhibiting primary and metastatic tumour growth, preventing tumour-recurrence, and extending survival through the induction of IFNg, inhibition of angiogenesis and increase in CTL activity 16. Likewise, a previous report from a similar veterinary clinical trial demonstrated that one cycle of ECGT with IL12 pDNA and bleomycin can safely and effectively treat large and small tumours of multiple histotypes 1,3. Most subjects to enter this trial had received previous treatments, such as radiation or surgery, so the tumours were recurrent, large or both. In such tumours, one cycle of any type of treatment is typically incapable of eliciting a complete response, but multiple cycles of ECGT with IL12 pDNA have yet to be studied.

To this end, 13 evaluable subjects with SCC, AA, or sarcomas received multiple treatment cycles of ECGT with IL12 and gemcitabine or bleomycin. In these studies, there were zero instances of severe adverse events at any dose level, and the only recurring minor adverse events were transient bleeding after treatment and ulceration because of necrosis of the tumour tissue, typically exacerbated by the canines' pre-dilection for licking the wound. Also, there were no instances of toxicity found in urinalysis or blood profiles (Table S1). This lack of severe adverse events is especially notable in the long-term cases.

Patients with very large, recurrent or multiple tumours generally require multiple treatment cycles to manage tumour growth. In several such patients, the safety of this EP-mediated system allowed for the safe administration of multiple cycles, and the versatility of EP-mediated treatments permitted response-based modifications to the treatments to reach multiple therapeutic endpoints within each patient while prolonging and improving the quality of life.

One SCC subject and two AA subjects (Figs. S2 and S3, Tables S3 and S4) received treatments over the course of 840, 240 and 280 days, respectively. Specifically, the recurrent SCC subject received 22 intratumoural treatments of IL12 pDNA plus chemotherapeutic drug through 12 treatment cycles with complete eradication of three separate tumours and without a single severe adverse event (Fig. S2, Table S3). Additionally, by switching the chemotherapeutic agent from bleomycin to gemcitabine and performing a dose escalation within the same subject, a progressive recurrent SCC lesion was eradicated after five treatments (three treatment cycles) and two dose escalations from 0.5 to 2 mg/cm. To the authors' knowledge, this is the first study to demonstrate the efficacy of switching chemotherapeutic agents with EP for eradicating chemo-resistant tumours. Further treatment in two locally recurrent SCC and the extramedullary PC with IL12 pDNA and increasing doses of gemcitabine inhibited tumour growth, although the tumours were not completely eradicated. Interestingly, the same treatment given concurrently in the two recurrent SCC (Fig. S2D, Lesions 4 and 5) resulted in different clinical responses. Since tumours are capable of developing different resistance to these treatments, the versatility of ECGT will allow for using different agents in multiple lesions within the same subject to specifically and efficiently treat each lesion. These results demonstrate the safety, efficacy and versatility of EP-mediated intratumoural treatments with IL12 pDNA.

In addition, these ECGT treatments are capable of quickly reducing tumour volume in large oral and cutaneous SCC lesions (Fig.4). These treatments reduced tumour volume by 37% in 21 days after only one cycle of ECGT treatments (Fig.4L). Likewise, repeated cycles of these treatments showed similar efficacy in SCC and PC, with gemcitabine and bleomycin being equally effective, but these treatments had no effect on sarcomas (Fig.1). Although these treatments did not inhibit or reduce sarcoma tumour growth, it is important to identify which histotypes do and do not respond to these treatments. Conversely, the EP-mediated IL12 pDNA only treatments did appear to stabilize sarcoma tumour growth (Fig.2C), and others have shown that IL12 therapy improves the efficacy of radiation in sarcomas 47 as well as ECT, but only when IL12 pDNA is administered at a distal site 48. Likewise, irreversible EP (IRE), a technique involving high-voltage electric pulses which directly kill tumour cells, can successfully treat sarcomas 35, and a recent study shows that IRE can also assist gene transfer allowing for gene treatments with IRE 34. So, although ECGT did not effectively treat sarcomas, IL12 pDNA may still yet be applicable to the treatment of sarcomas.

One of the benefits to including the IL12 pDNA is the induction of a robust yet specific antitumour response 27,36–39. All tumours in the few subjects to receive IL12 pDNA only treatments in this trial responded well with no PDs (Fig.2, Table2). Also, none of the SCC tumours located on the nasal planum responded to ECGT treatments (Fig.2E, Table2), although others have reported successful treatment with ECT alone (no IL12 pDNA) of tumours in felines. Interestingly, nasal planum tumours have a reduced level of infiltrating T cells 49, and this reduction in immune access may reduce the efficacy of the immunomodulatory portion of the ECGT treatments 50. Notably, a non-treated metastatic lymph node tumour decreased in volume by almost 80% after only one cycle of treatments (Fig.4D). In agreement with this observation, ECGT has been previously shown to induce an immune response which completely eradicated a non-treated metastatic bone tumour 1. In addition, rtPCR analysis of IFNg and CD8 mRNA expression in treated tumours revealed a significant fourfold increase in IFNg levels in responding tumours, while IFNg levels in non-responding tumours did not change (Fig. S4A). CD8 was also increased in responding tumours, but this difference was not statistically significant (Fig. S4B). Furthermore, these treatments equally affect small and large tumours (Fig.3), such as those for which ECT without IL12 is not as effective 46. Together, these data demonstrate the importance of the immunomodulatory effect of the IL12 in these ECGT treatments.

All of the data presented thus far has demonstrated that ECGT with IL12 and bleomycin or gemcitabine is safe, well-tolerated, effective and versatile, but the effects have yet to be compared to standard therapies. For SCC and most other cancers, surgery is the best option if available and would not detract from the quality of life. In canines, surgery (amputation of affected limb or minimum 2-cm surgical margins) confers a 1-year survival of 95% and a median survival time of 474 days. On the other hand, the 1-year survival rate for invasive SCC with surgery is only 10%. To identify the benefit of ECGT compared to standard, non-surgical treatment options, the survival time of subjects with complete medical records treated with radiation or chemotherapy at GCVS, the host of this trial, were compared to all evaluable subjects treated with at least 1 cycle of ECGT in this trial. The median survival for subjects receiving standard therapy was only 97 days compared to 287 days for ECGT, a 3-fold extension of survival. Likewise, the 1-year survival was nearly 25% for ECGT compare to only 17% for standard therapy (Fig.1E). So, surgery, if not debilitating, remains the best option, but ECGT should become a first-line treatment based on the prolonged survival with these treatments.

These exciting results highlight the versatility, safety, tolerability and efficacy of repeated administration of IL12 pDNA, chemotherapy and EP. ECGT with IL12 pDNA plus bleomycin or gemcitabine can safely and effectively diminish target and non-target lesions in SCC, PC and AA, but not sarcomas. In oral and cutaneous SCC, these ECGT treatments can eradicate large and small tumours or significantly reduce the volume to improve the quality of life and possibly enhance the efficacy of subsequent surgery, radiation or other therapies. In addition, these results further show the potential of immunomodulatory cancer treatments and will help to finally turn this potential into a reality.

## Supporting Information

**Figure S1.** Eradication of SCC tumours by ECGT with IL12 pDNA and gemcitabine in a murine model.

**Figure S2.** Long-term management of multiple tumours in a single subject with ECGT.

**Figure S3.** Tumour volume graph representing the entirety of the treatments for both Acanthomatous Ameloblastoma patients.

**Figure S4.** IFNg and CD8 mRNA levels are increased in tumours of responding tumours and not non-responding tumours.

**Table S1.** Details of treatment cycles for SCC patient described in Figure . **Table S2.** Example of CBC and blood chemistry analysis from the patient described in Figure  and Table S1 demonstrating the safety of ECGT treatments. **Table S3.** Details of Treatments for Acanthomatous Ameloblastoma patient 1 (Fig. S2A). **Table S4.** Details of Treatments for Acanthomatous Ameloblastoma patient 2 (Fig. S2B).

## References

[b1] Cutrera J, Torrero M, Shiomitsu K, Li S (2008). Intratumoral bleomycin and IL-12 electrochemogenetherapy for treating head and neck tumors in dogs. Electroporation protocols: preclinical and clinical gene medicine.

[b2] Kishida T, Asada H, Itokawa Y (2003). Electrochemo-gene therapy of cancer: intratumoral delivery of interleukin-12 gene and bleomycin synergistically induced therapeutic immunity and suppressed subcutaneous and metastatic melanomas in mice. Mol Ther.

[b3] Reed SD, Fulmer A, Buckholz J (2010). Bleomycin/interleukin-12 electrochemogene therapy for treating naturally occurring spontaneous neoplasms in dogs. Cancer Gene Ther.

[b4] Adelstein DJ (2003). Systemic chemotherapy for squamous cell head and neck cancer. Exp Opin Pharmacother.

[b5] DeConti RC (2012). Chemotherapy of squamous cell carcinoma of the skin. Semin Oncol.

[b6] Chuang TF, Lee SC, Liao KW (2009). Electroporation-mediated IL-12 gene therapy in a transplantable canine cancer model. Int J Cancer.

[b7] Cohen J (1995). IL-12 deaths: explanation and a puzzle. Science.

[b8] Campana LG, Mocellin S, Basso M (2009). Bleomycin-based electrochemotherapy: clinical outcome from a single institution's experience with 52 patients. Ann Surg Oncol.

[b9] Kodre V, Cemazar M, Pecar J (2009). Electrochemotherapy compared to surgery for treatment of canine mast cell tumours. In Vivo.

[b10] Spugnini EP, Dotsinsky I, Mudrov N (2008). Successful rescue of an apocrine gland carcinoma metastatic to the cervical lymph nodes by mitoxantrone coupled with trains of permeabilizing electrical pulses (electrochemotherapy). In Vivo.

[b11] Spugnini EP, Filipponi M, Romani L (2007). Local control and distant metastasis after electrochemotherapy of a canine anal melanoma. In Vivo.

[b12] Tozon N, Kodre V, Sersa G (2005). Effective treatment of perianal tumors in dogs with electrochemotherapy. Anticancer Res.

[b13] Spugnini EP, Vincenzi B, Citro G (2011). Evaluation of cisplatin as an electrochemotherapy agent for the treatment of incompletely excised mast cell tumors in dogs. J Vet Intern Med.

[b14] Miklavcic D, Sersa G, Brecelj E (2012). Electrochemotherapy: technological advancements for efficient electroporation-based treatment of internal tumors. Med Biol Eng Comput.

[b15] Daud AI, DeConti RC, Andrews S (2008). Phase I trial of interleukin-12 plasmid electroporation in patients with metastatic melanoma. J Clin Oncol.

[b16] Torrero MN, Henk WG, Li S (2006). Regression of high-grade malignancy in mice by bleomycin and interleukin-12 electrochemogenetherapy. Clin Cancer Res.

[b17] Pavlin D, Cemazar M, Sersa G (2012). IL-12 based gene therapy in veterinary medicine. J Transl Med.

[b18] Buttner M, Belke-Louis G, Rziha HJ (1998). Detection, cDNA cloning and sequencing of canine interleukin 12. Cytokine.

[b19] Okano F, Satoh M, Yamada K (1997). Cloning and expression of the cDNA for canine interleukin-12. J Interferon Cytokine Res.

[b20] Cutrera J, King G, Jones P, Li S, Cutrera J, Heller R, Teissie J (2014). Managing local swelling following intratumoral electro-chemo-gene therapy. Electroporation protocols: preclinical and clinical gene medicine.

[b21] Cutrera J, Dibra D, Xia X (2011). Discovery of a linear peptide for improving tumor targeting of gene products and treatment of distal tumors by IL-12 gene therapy. Mol Ther.

[b22] Li S, Zhang L, Torrero M (2005). Administration route- and immune cell activation-dependent tumor eradication by IL12 electrotransfer. Mol Ther.

[b23] Escoffre JM, Rols MP (2012). Electrochemotherapy: progress and prospects. Curr Pharm Des.

[b24] Mir LM (2006). Bases and rationale of the electrochemotherapy. EJC Suppl.

[b25] Frese KK, Neesse A, Cook N (2012). nab-Paclitaxel potentiates gemcitabine activity by reducing cytidine deaminase levels in a mouse model of pancreatic cancer. Cancer Discov.

[b26] Atkins MB, Robertson MJ, Gordon M (1997). Phase I evaluation of intravenous recombinant human interleukin 12 in patients with advanced malignancies. Clin Cancer Res.

[b27] Del Vecchio M, Bajetta E, Canova S (2007). Interleukin-12: biological properties and clinical application. Clin Cancer Res.

[b28] Cutrera J, Li S, Lustgarten J, Cui Y, Li S (2009). Passive and active tumor homing cytokine therapy. Targeted cancer immune therapy.

[b29] Wei LZ, Xu Y, Nelles EM (2013). Localized interleukin-12 delivery for immunotherapy of solid tumours. J Cell Mol Med.

[b30] Li S, Cutrera J, Teissie J, Heller R (2014). Electroporation protocols: preclinical and clinical gene medicine.

[b31] Teissie J, Li S, Cutrera J, Heller R, Teissie J (2014). Electropermeabilization of the cell membrane. Electroporation protocols: preclinical and clinical gene medicine.

[b32] Heller L, Merkler K, Westover J (2006). Evaluation of toxicity following electrically mediated interleukin-12 gene delivery in a B16 mouse melanoma model. Clin Cancer Res.

[b33] Spugnini EP, Dotsinsky I, Mudrov N (2008). Biphasic pulses enhance bleomycin efficacy in a spontaneous canine genital tumor model of chemoresistance: Sticker sarcoma. J Exp Clin Cancer Res.

[b34] Au JT, Mittra A, Song TJ (2013). Irreversible electroporation facilitates gene transfer of a GM-CSF plasmid with a local and systemic response. Surgery.

[b35] Neal R, Rossmeisl J, Garcia P (2011). Successful treatment of a large soft tissue sarcoma with irreversible electroporation. J Clin Oncol.

[b36] Motzer RJ, Rakhit A, Schwartz LH (1998). Phase I trial of subcutaneous recombinant human interleukin-12 in patients with advanced renal cell carcinoma. Clin Cancer Res.

[b37] Gautam SC, Xu YX, Dumaguin M (2000). Interleukin-12 (IL-12) gene therapy of leukemia: immune and anti-leukemic effects of IL-12-transduced hematopoietic progenitor cells. Cancer Gene Ther.

[b38] Heinzerling LM, Feige K, Rieder S (2001). Tumor regression induced by intratumoral injection of DNA coding for human interleukin 12 into melanoma metastases in gray horses. J Mol Med.

[b39] Yamazaki M, Zhang R, Straus FH (2002). Effective gene therapy for medullary thyroid carcinoma using recombinant adenovirus inducing tumor-specific expression of interleukin-12. Gene Ther.

[b40] Muller J, Feige K, Wunderlin P (2011). Double-blind placebo-controlled study with interleukin-18 and interleukin-12-encoding plasmid DNA shows antitumor effect in metastatic melanoma in gray horses. J Immunother.

[b41] Muller JM, Wissemann J, Meli ML (2011). *In vivo* induction of interferon gamma expression in grey horses with metastatic melanoma resulting from direct injection of plasmid DNA coding for equine interleukin 12. Schweiz Arch Tierheilkd.

[b42] Pavlin D, Cemazar M, Cor A (2011). Electrogene therapy with interleukin-12 in canine mast cell tumors. Radiol Oncol.

[b43] Li S (2008). Electroporation protocols: preclinical and clinical gene medicine.

[b44] Tozon N, Sersa G, Cemazar M (2001). Electrochemotherapy: potentiation of local antitumour effectiveness of cisplatin in dogs and cats. Anticancer Res.

[b45] Spugnini EP, Baldi A (2014). Electrochemotherapy in veterinary oncology: from rescue to first line therapy. Methods Mol Biol.

[b46] Mali B, Miklavcic D, Campana L (2013). Tumor size and effectiveness of electrochemotherapy. Radiol Oncol.

[b47] Sedlar A, Kranjc S, Dolinsek T (2013). Radiosensitizing effect of intratumoral interleukin-12 gene electrotransfer in murine sarcoma. BMC cancer.

[b48] Sedlar A, Dolinsek T, Markelc B (2012). Potentiation of electrochemotherapy by intramuscular IL-12 gene electrotransfer in murine sarcoma and carcinoma with different immunogenicity. Radiol Oncol.

[b49] Vanherberghen M, Day MJ, Delvaux F (2009). An immunohistochemical study of the inflammatory infiltrate associated with nasal carcinoma in dogs and cats. J Comp Pathol.

[b50] Quetglas JI, Hervas-Stubbs S, Smerdou C (2013). The immunological profile of tumor-bearing animals determines the outcome of cancer immunotherapy. Oncoimmunology.

