# Supplementary figures and images for: Safe and effective treatment of spontaneous neoplasms with interleukin 12 electro-chemo-gene therapy
Source: J Cell Mol Med. 2015 Jan 27;19(3):664–75. doi: 10.1111/jcmm.12382 (PMC4369822; doi:10.1111/jcmm.12382)

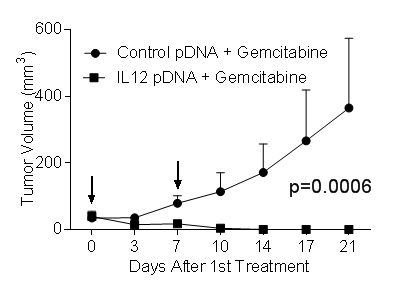

Supplement: Supplementary file 1 [file jcmm0019-0664-sd1.tif]

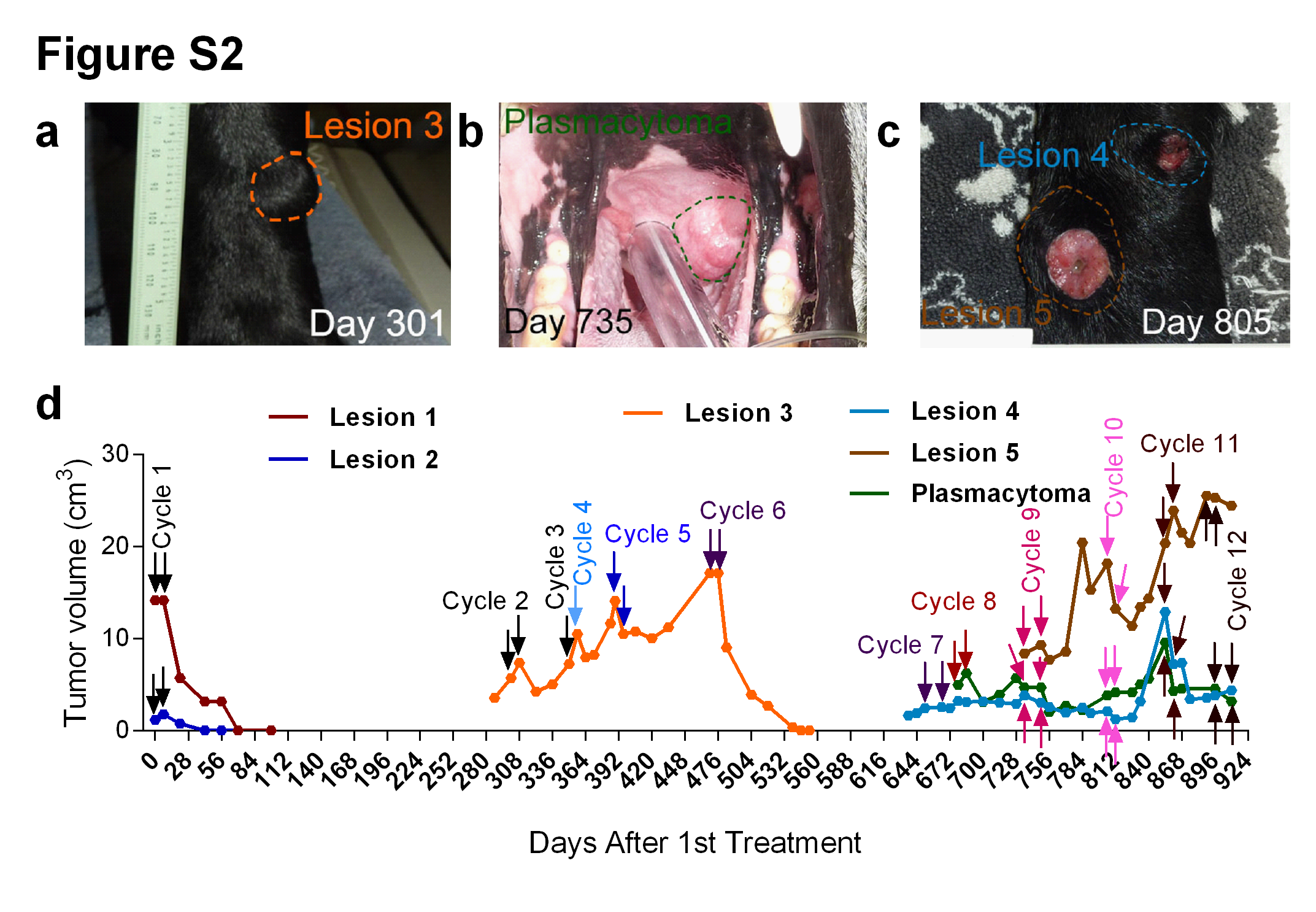

Supplement: Supplementary file 2 [file jcmm0019-0664-sd2.tif]

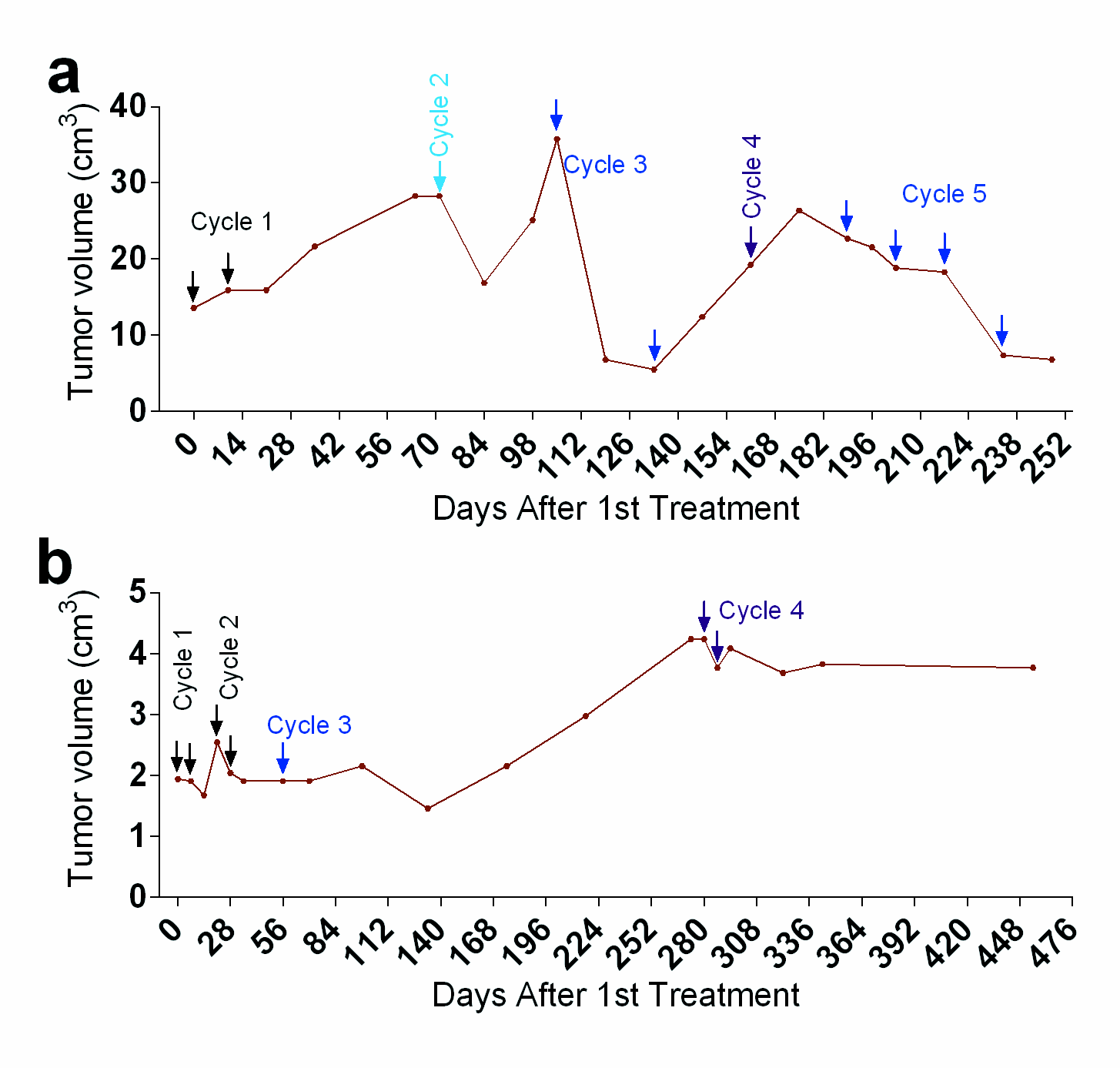

Supplement: Supplementary file 3 [file jcmm0019-0664-sd3.tif]

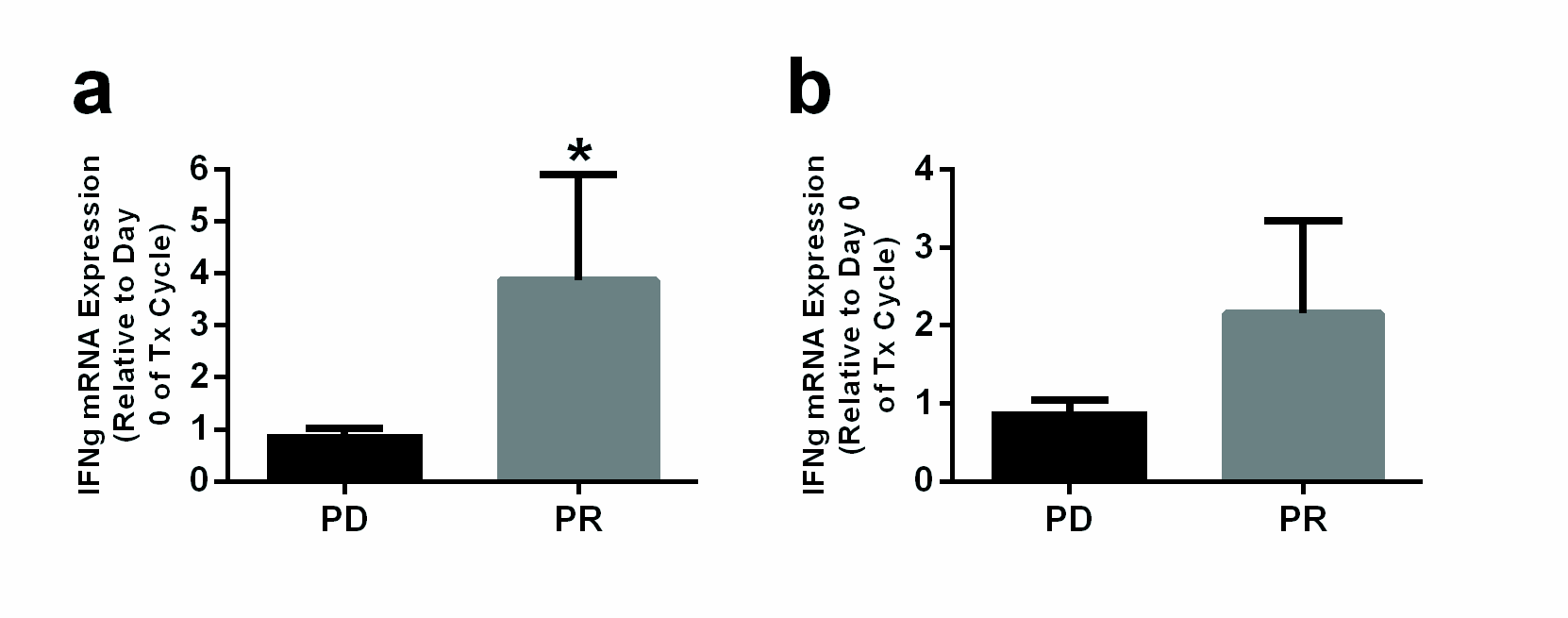

Supplement: Supplementary file 4 [file jcmm0019-0664-sd4.tif]
