# Supplementary material for: Safe and effective treatment of spontaneous neoplasms with interleukin 12 electro-chemo-gene therapy
Source: J Cell Mol Med. 2015 Jan 27;19(3):664–75. doi: 10.1111/jcmm.12382 (PMC4369822; doi:10.1111/jcmm.12382)
Supplement: Supplementary file 5 [file jcmm0019-0664-sd5.doc]

**Supplementary Figure Captions**

**Figure S1.** Eradication of SCC tumors by ECGT with IL12 pDNA and gemcitabine in a murine model. Only intratumoral ECGT treatment with gemcitabine and IL12 pDNA, and not control pDNA, can eradicate SCCVII tumors in C3H mice (n=5).

**Figure S2.** Long-term management of multiple tumors in a single subject with ECGT. This canine subject received 22 treatments over the course of 12 treatment cycles with different clinical responses. After only 1 cycle (Cycle 1) of ECGT with IL12 pDNA and bleomycin, both initial recurrent squamous cell carcinoma tumors gradually regressed. On day 301, another recurrent SCC nodule (**a**, Lesion 3) was noted in a similar location. Subsequent treatments of ECGT with IL12 pDNA and dose-escalating gemcitabine eventually resulted in another complete response (Cycle 4-6). Additional cycles of treatment were not capable of completely eradicating the subsequent recurrent SCC nodules (**c**, Lesions 4 and 5) nor the oral plasmacytoma (**b**, Plasmacytoma). Full details for the treatments of each cycle can be found in **Table S1**. **d** The complete treatment timeline.

**Figure S3.** Tumor volume graph representing the entirety of the treatments for both Acanthomatous Ameloblastoma patients. **a** Patient 1: Day 0 through Day 252. Arrows denote treatments, and the colors of the arrows represent the different treatments. The black arrows denote IL12 plus bleomycin (Cycle 1), the light blue arrow denotes IL12 and bleomycin separate treatments (Cycle 2), the dark blue arrows represents the IL12 only treatments (Cycles 3 and 5), and the purple arrow denotes IL12 alone in the caudal area and IL12 plus bleomycin in the rostral area (Cycle 4). **b** Patient 2: Day 0 through Day 455. The black arrows denote IL12 pDNA only treatments (Cycles 1 and 2), the blue arrow represents the IL12 plus bleomycin treatment of Cycle 3, and the purple arrows denote IL12 pDNA plus gemcitabine treatments (Cycle 4).

**Figure S4.** IFNg and CD8 mRNA levels are increased in tumors of responding tumors and not non-responding tumors. **a** Biopsies from tumors on 14 after EP-mediated treatments show a 4-fold increase in IFNγ expression compared to Day 0 levels in responding tumors (n=4), but no increase in IFNγ expression in progressive tumors (n=3). **b** A 2-fold increase is seen in CD8 expression, however, this difference is not significant.

**Supplementary Tables**

Table S1. Details of treatment cycles for SCC patient described in Figure 1. Blm, bleomycin; Gem, gemcitabine; CR, complete response; PR, partial response; SD, stable disease; PD, progressive disease.

| Treatment Cycle  (Color in Figures) | # of Treatments per Round (Interval) | Chemo Drug | # of Injections per Treatment | Chemo per Injection (mg) | Total Chemo per Treatment (mg) | Lesions Treated | Days (after 1st Treatment) | Therapeutic Response |
| --- | --- | --- | --- | --- | --- | --- | --- | --- |
| 1  (Black) | 2  (7 days) | Blm | 6 | 1 | 6 | Lesion 1 and 2 | 0,7 | CR |
| 2  (Black) | 2  (7 days) | Blm | 3 | 1 | 3 | Lesion 3 | 301,308 | PD |
| 3  (Black) | 1 | Blm | 3 | 1 | 3 | Lesion 3 | 350 | PD |
| 4  (Light Blue) | 1 | Gem | 3 | 1 | 3 | Lesion 3 | 357 | PD |
| 5  (Blue) | 2  (7 days) | Gem | 3 | 2 | 6 | Lesion 3 | 389, 396 | PR |
| 6  (Purple) | 2  (7 days) | Gem | 4 | 4 | 16 | Lesion 3 | 476, 483 | CR |
| 7  (Purple) | 2  (14 days) | Gem | 3 | 4 | 12 | Lesion 4 | 651, 665 | PR |
| 8  (Red) | 2  (7 days) | Gem | 2 | 2 | 4 | Plasma Cell | 679, 686 | PD |
| 9  (Fuschia) | 2  (14 days) | Gem | 6 | 8 | 48 | Lesion 4, 5, and Plasma Cell | 735, 749 | SD, PD,  and PR |
| 10  (Pink) | 2  (7 days) | Gem | 8 | 10 | 80 | Lesion 4, 5, and Plasma Cell | 805, 812 | SD, PD,  and SD |
| 11  (Brown) | 2  (7 days) | Blm | 7 | 1 | 7 | Lesion 4, 5, and Plasma Cell | 854, 861 | SD, PR,  and PR |
| 12  (Dark Brown) | 2  (7 days) | Blm | 7 | 2 | 14 | Lesion 4, 5, and Plasma Cell | 854, 861 | SD, SD,  and SD |

Table S2. Example of CBC and blood chemistry analysis from the patient described in Figure 1 and Table S1 demonstrating the safety of ECGT treatments.

|  |  | Prior to Treatment | | After Treatment | |
| --- | --- | --- | --- | --- | --- |
| Test | Reference | Results | Quality | Results | Quality |
| RBC (M/µL) | 5.4-7.8 | 8.12 | High | 6.95 | Normal |
| HCT (%) | 37.0-54.0 | 46.70 | Normal | 47.90 | Normal |
| HGB (g/dL) | 13.0-19.0 | 16.20 | Normal | 16.20 | Normal |
| MCV (fL) | 66.0-75.0 | 57.50 | Low | 68.90 | Normal |
| MCH (pg) | 22.00-27.00 | 20.00 | Low | 23.30 | Normal |
| MCHC (g/dL) | 30.0-37.5 | 34.70 | Normal | 33.80 | Normal |
| RDW (%) | 11.0-23.0 | 24.80 | High | 19.40 | Normal |
| %RETIC (%) |  | 1.10 | Normal | 0.40 | Normal |
| RETIC (K/µL) | 0.0-60.0 | 86.10 | High | 27.10 | Normal |
| WBC (K/µL) | 6.00-17.00 | 6.76 | Normal | 7.97 | Normal |
| NEU (K/µL) | 3.90-8.00 | 5.22 | Normal | 5.19 | Normal |
| LYM (K/µL) | 1.30-4.10 | 0.63 | Normal | 1.76 | Normal |
| MONO (K/µL) | 0.20-1.10 | 0.68 | Normal | 0.48 | Normal |
| EOS (K/µL) | 0.00-0.60 | 0.22 | Normal | 0.52 | Normal |
| BASO (K/µL) | 0.00-0.10 | 0.01 | Normal | 0.02 | Normal |
| PLT (K/µL) | 150-430 | 353.00 | Normal | 189.00 | Normal |
| MPV (fL) | 8.00-16.00 | 9.10 | Normal | 10.70 | Normal |
| PDW (fL) |  | 11.00 | Normal | 13.50 | Normal |
| PCT (%) |  | 0.30 | Normal | 0.20 | Normal |

Table S3. Details of Treatments for Acanthomatous Ameloblastoma patient 1 (Figure S2a). Blm, bleomycin; PR, partial response; PD, progressive disease. pDNA, plasmid DNA; IL12, Interleukin 12

| Treatment Cycle  (Color in Figures) | # of Treatments per Round (Interval) | Treatments Administered | Tumor Area Treated | Days (after 1st Treatment) | Therapeutic Response |
| --- | --- | --- | --- | --- | --- |
| 1  (Black) | 2  (7 days) | IL12 pDNA plus blm | All | 0,10 | PD |
| 2  (Light Blue) | 1 | IL12 pDNA; blm | Rostral; Caudal | 70 | PR |
| 3  (Dark Blue) | 2  (28 days) | IL12 pDNA | Caudal | 105, 133 | PR |
| 4  (Purple) | 1 | IL12 pDNA; IL12 pDNA plus blm | Rostral; Caudal | 161 | PD |
| 5  (Dark Blue) | 4  (14 days) | IL12 pDNA | All | 182, 203, 217, 231 | PR |

Table S4. Details of Treatments for Acanthomatous Ameloblastoma patient 2 (Figure S2b). Blm, bleomycin; Gem, gemcitabine; SD, stable disease; PD, progressive disease. pDNA, plasmid DNA; IL12, Interleukin 12

| Treatment Cycle  (Color in Figures) | # of Treatments per Round (Interval) | Treatments Administered | Days (after 1st Treatment) | Therapeutic Response |
| --- | --- | --- | --- | --- |
| 1  (Black) | 2  (7 days) | IL12 pDNA | 0,7 | PD |
| 2  (Black) | 2  (7 days) | IL12 pDNA | 21, 28 | SD |
| 3  (Blue) | 1 | IL12 pDNA plus blm | 56 | SD |
| 4  (Purple) | 2  (7 days) | IL12 pDNA plus Gem | 280, 287 | SD |
